# Supplementary material for: Are carbon emissions trading and green financial instruments synergistic? -Comprehensive quantitative research based on content analysis
Source: PLoS One. 2024 Mar 7;19(3):e0298601. doi: 10.1371/journal.pone.0298601 (PMC10919601; doi:10.1371/journal.pone.0298601)
Supplement: S1 Data — (ZIP) [file pone.0298601.s001.zip › Data/地级市Z_F工作报告.zip!/╝¬┴╓/│ñ┤║╩╨/└┤╫╘╣1⁄2╓┌║┼í╛┬φ┐╦╩2╛▌═°í┐_2005.docx]

各位代表：

　　我代表市人民政府向大会作工作报告，请予审议。并请列席会议的同志提出意见。

　　一、2004年工作简要回顾

　　过去的一年，我们在省委、省政府和市委的领导下，在市人大、政协的监督和支持下，认真贯彻党的十六大和十六届三中、四中全会精神，树立和落实科学发展观，紧紧依靠全市人民，积极推进改革开放和现代化建设，较好地完成了全年各项工作任务和目标，实现了长春老工业基地振兴的良好开局。

　　（一）国民经济持续快速增长，发展后劲进一步增强。预计实现全市生产总值1535亿元，比上年增长13.5%。实现全口径财政收入154.2亿元，增长14.3%。农业生产喜获丰收。粮食产量达到160亿斤，增产26亿斤。一产实现增加值168亿元，增长8%。克服禽流感影响，畜牧业实现增加值91亿元，增长12%。农民外出务工经商85万人次，增加10万人次。工业持续平稳增长，实现增加值635亿元，增长14.7%。规模以上工业实现产值1712亿元，增长13.4%。汽车工业在竞争中稳步发展，实现产值1336.4亿元，增长15.8%。食品、光电子信息、生物与医药产业增势良好，产值分别增长30.4%、36.7%和33.4%。建筑业实现增加值107亿元，增长20%。会展旅游业发展迅速，举办各类展会100余项，带动相关行业收入56亿元。商贸物流、信息中介发展势头良好，实现全社会消费品零售总额495亿元，增长13%。房地产市场日趋活跃，实现房屋交易470万平方米、交易额100亿元，分别增长22.7%和33.8%。防范和化解地方金融风险取得显著成效，市商业银行首次实现年度盈利，农村信用社净增股本金6.6亿元，累计发行的11.6亿元地方企业债券全部兑付。

　　固定资产投资在土地和信贷政策收紧情况下，实现稳步增长。完成全社会固定资产投资460亿元，增长18%。投资结构不断优化，非国有投资增长37%，占全部投资的55%，提高4个百分点。新建续建3000万元以上投资项目308个，其中超10亿元的21个。一汽“三大基地”、皓月肉牛综合开发、大成姚家玉米、德莱鹅肥肝、九台金锣肉制品等一批骨干项目部分或全部建成投产，汽车、农产品等重点产品生产和加工能力大幅度提高。争取国债项目取得明显成效，21个工业项目和18个高技术项目列入国家计划，总投资128.7亿元。积极打造新的项目建设载体，长春光电子国家产业基地正式得到批准，长春生物医药国家高技术产业基地、国家汽车零部件出口基地申报工作进展顺利。

　　（二）各项改革扎实推进，对外开放取得新成效。91户国有企业基本完成改制，1.9万名国企职工理顺了劳动关系。私营企业和个体工商户分别新增2563户和29594户，民营经济实现增加值535亿元，实缴税金28.8亿元，均增长20%。农村税费改革顺利实施，“一免三补”政策全面落实，农民人均政策性增收230元。粮食流通体制改革全面展开。完成了新一轮政府机构改革，国有资产监管、安全生产监管、维护城市安全和为中小企业发展服务的职能进一步增强。精心开展招商引资工作。先后9次组织赴日、韩、德、新加坡、香港、“长三角”、“珠三角”等国家和地区开展大型招商活动，开展了韩国周、香港周等经贸交流活动，接待国内外经贸考察团组231个，对外签约项目684个，新批外商投资项目127个,世界500强企业又有李尔等9家落户长春，总数达到38家。合同利用外资12.8亿美元，增长2.3倍。实际利用外资9亿美元，增长20%。引进内资项目454个，实际利用内资166亿元，增长14.5%。开发区经济稳步增长，实现生产总值712亿元，占全市比重达到46%。外贸出口商品结构不断优化，高新技术和机电产品出口分别增长40%和66%。长春空港落地签证获得国家批准，建立了对外交流与合作的便捷通道。倡议并成功举办东北四城市市长峰会，促进了区域经济合作，扩大了对外影响。

　　（三）人民生活水平进一步提高，就业和社会保障体系不断完善。城市居民人均可支配收入达到8900元，增长12.6%。农民人均纯收入达到3906元，增幅达14.5%。居民消费结构不断升级。每万户城市居民新增私人小汽车113辆、电脑1000台，城镇人均住房建筑面积增加到23.1平方米。农村居民恩格尔系数下降0.4个百分点，程控电话、彩电、洗衣机普及率分别达到48%、79%和70%。

　　全面落实税费优惠、小额贷款等再就业扶持政策，为下岗失业人员减免税费和提供补贴4794万元，2300名残疾人、1.3万名“4050”人员、6.5万名下岗失业人员实现就业和再就业，1100人实现成功创业，城镇登记失业率为3.96%，被评为全国再就业工作先进城市。社会保障能力进一步增强。养老、失业、医疗保险参保人数分别新增8万人、4万人和12.8万人。征缴养老、失业保险基金22.7亿元，27.5万名离退休人员养老金和3.3万名失业人员失业金得到按时、足额发放。完善社会保障体系试点工作顺利启动，做实职工养老保险个人账户准备工作基本完成。进一步加强企业退休人员动态管理，社会化管理率达到97.2%。落实廉租房、粮油补贴、采暖费减免等配套政策，为低保家庭补贴2200余万元，1017户低保家庭住房问题得到解决。社会救助工作深入开展，各级财政投入4927万元，救助农村受灾群众8.4万人次、其他困难群众20余万人次。

　　切实解决群众关心的热点、难点问题。改造城区供水管线101公里、燃气管线103公里、二次供水设施400处，新增集中供热面积250万平方米。千方百计组织煤炭供应，居民供暖及时开栓率达到95%。1452户超期回迁居民得到安置，6000户煤气遗留问题和8200户临时用电问题得到解决。改造城区巷道146条，亮化背街小巷221条。新建和改造城区公厕207座。新建乡村水泥（油）路2841公里，通油路行政村比例达到73%。改造洼中高引水工程主管线17.5公里，农安氟病区新打防氟改水井100眼。认真处理群众来信来访，落实信访责任制和责任追究制，249件信访难点问题得到解决。加强社会治安综合治理，严厉打击各类刑事犯罪，刑事案件发案率得到有效控制。强化安全生产责任制，杜绝了特大安全事故发生，安全生产事故下降19%。

　　（四）城市建设步伐加快，管理水平进一步提高。开展了城市空间发展战略规划、新一轮城市总体规划和土地利用总体规划修编，“长春国际汽车城”等13个专项和分区规划编制工作顺利启动。新建道路16条，大中修和维护道路369条，长吉南线等6条公路竣工通车。科铁线等3条公路完成年度施工计划。积极开展轻铁二期工程立项申报工作，通过了国家发改委和建设部审批，并上报国务院待批。石头口门水库兴利增容全面完成，热电二厂二期改造等工程进展顺利。波罗湖湿地生态恢复、“三北”四期防护林工程步伐加快，南湖公园、胜利公园改造顺利推进。完成人工造林7100公顷，城区新增绿地303公顷。治理不合格锅炉335台（套），整治违法排污企业401家，环境质量进一步改善。取缔马路市场和占道经营76处，依法对街路牌匾、非法广告、违章占道、非法挖掘道路和城市出入口进行了集中整治，城市管理进一步加强。清理整顿土地市场，经营性用地全面实行招标、拍卖和挂牌出让，出让土地344公顷，土地资源利用效率进一步提高。

　　（五）精神文明建设不断深入，各项社会事业全面发展。全国文明城市等创建活动深入开展，市民的文明素质进一步提高。广泛开展“全民创业、振兴长春”实践活动，大力培育创业文化，全民创业氛围更加浓厚。文化事业发展较快，成功举办了第六届亚洲艺术节、第七届国际萨满文化学术研讨会和第九届长春书市，开展优秀剧目公益性巡回演出80余场，组织各类文艺活动1000余场（次），丰富、活跃了群众的文化生活。文化基础设施建设得到加强，伪满皇宫三年复原等工程顺利完成，市图书馆被评为国家一级公共图书馆。科技创新体系不断完善，企业技术中心发展到100户，制造业信息化示范企业发展到120户。自主创新能力进一步提升，申请发明专利815件，增长18.7%。高新技术产业化进程加快，实现产值650亿元，增长25%，被评为全国科技进步先进城市。“人才强市”战略扎实推进，引进高层次急需和紧缺人才1万多人，培训农村实用人才4万多人次。认真办理人大关于发展农村教育的议案，对农村教育的扶持力度进一步加大。3.8万名农村贫困学生得到救助，2万名乡村教师得到免费培训，276所农村薄弱校得到社会的对口帮扶，9.3万平方米农村中小学危倒校舍得到改造。“教育质量年”活动成效明显，城区优质高中招生比例达到86.9%，高考本科进线率提高10%。职业教育招生与就业两旺，招生增长25%，毕业生一次性就业率保持在96%以上。民族教育得到加强，少数民族学校办学条件、教学质量进一步改善。制定了《重大急性传染病疫情应急处理预案》，重新整合了“120”急救医疗网络，新建的市、县两级疾控中心相继交付使用，应对公共卫生突发事件能力明显提高。九台新型农村合作医疗制度试点初见成效，医药“三网”建设进展顺利，农村医疗卫生条件得到初步改善。亚冬会筹备工作稳步推进，确定了会徽、吉祥物，争得了市场开发权和电视转播权，五环体育馆、市滑冰馆配套设施部分完工，莲花山滑雪场建成并投入使用。人口与计划生育工作取得新进展，被国家评为人口与计划生育综合改革一流城市。全市351个社区服务用房建设全面完成，平均建筑面积达到550平方米，位居全国大中城市前列。60个“阳光超市”建成并投入使用，社区民主自治与服务功能不断加强。以科技拥军为重点的拥军优属、拥政爱民工作成效显著，援藏工作取得新进展。妇女儿童、广播电视、人防、预备役、民兵、民族宗教、侨务、老龄、残联、地震、档案和修志等工作也都取得了新成绩。

　　（六）民主法制建设稳步推进，政府自身建设进一步加强。自觉接受人大及其常委会的法律监督、工作监督和政协的民主监督，接受各级人大代表和政协委员视察、检查52次。直接面复成为办理人大议案、建议的主要形式，除极特殊原因均做到面复。办理人大议案、建议201件，办理政协建议案、提案323件，办结率均达到100%。提请市人大常委会审议通过地方性法规8件，制定政府规章及规范性文件23件。依法治市工作得到加强，政府依法行政意识明显提高。认真贯彻《行政许可法》，清理行政许可519项，其中我市自行设定的80项按规定保留7项。政务中心功能进一步完善，行政许可办理基本做到“一个窗口进出”。政务公开进一步深化，50个部门实行网上公开，155处公共场所设立了政务信息公开查询点。组建了决策咨询委员会，政府决策的科学化、民主化水平进一步提高。建立新闻发言人制度，在市直57个部门设立了新闻发言人，组织各类新闻发布会41次。电子政务建设得到加强，构建了全市统一的电子政务网络平台，部门间实现互联互通。成立了软环境投诉举报中心，受理涉软案件1313件，办结1272件，给予党政纪处分67人。清费减负工作深入开展，先后清理132个收费项目，为企业和消费者减负1.3亿元。组织政府采购公开招投标412次，节约财政资金6123万元。认真倾听群众呼声，市长公开电话业务拓展到媒体和社区，共办理群众来电15.2万件，办结率达到95.8%。开展群众建议征集活动，共征集建议2613条，采纳468条。

　　上述成绩的取得，是市委正确领导的结果，是人大、政协监督和支持的结果，是全市人民不懈努力的结果，是社会各界通力合作、广泛参与的结果。在此，我代表市人民政府，向为长春发展做出无私奉献、付出辛勤劳动的全市人民，表示崇高敬意！向给予政府工作大力支持的人大代表和政协委员，向各民主党派、工商联、无党派人士和社会团体，向人民解放军驻长部队和武警官兵，向中央和省驻长单位，向所有关心、支持长春发展的海内外朋友们，表示诚挚的感谢！

　　在充分肯定成绩的同时，我们也清醒地看到，我市经济社会和政府工作还存在许多亟待解决的矛盾和问题。主要表现在：经济的结构性矛盾突出，整体抗风险、抗波动能力较弱；固定资产投资增速不高；民营经济规模小，发展活力不足；地方可用财力偏低，支撑改革、发展和稳定的能力不强；县域经济发展不快；城市基础设施欠账较多，社会公共安全体系建设任务繁重；城市安全隐患、社会不安定因素仍然较多，就业和社会保障、维护社会稳定压力增大；政府运行机制和自身建设有待进一步加强，部分工作人员素质不高、服务意识不强，个别人行政不公、为政不廉，甚至以权谋私、贪污腐败。对于这些问题，我们一定高度重视，并切实加以解决。

　　二、2005年主要任务

　　2005年是“十五”计划的最后一年，也是继续实施老工业基地振兴战略、推动各项工作再上新台阶的关键一年。长春既拥有巨大的发展潜力和难得机遇，又面临各种困难和严峻挑战。能否抓住新机遇、开拓新思路、采取新举措、解决新问题、实现新发展，是对我们的重大考验。我们一定坚持加快发展不动摇，以创新的精神、扎实的作风、百倍的努力，全力推动我市经济和各项社会事业快速发展。

　　今年政府工作的总体思路是：以邓小平理论和“三个代表”重要思想为指导，深入贯彻党的十六大、十六届三中、四中全会、中央经济工作会议和省委八届六次全会、全省经济工作会议、市委十届六次全会精神，全面落实科学发展观，进一步提高行政能力，围绕振兴长春老工业基地，加快推进改革开放，加快转变经济增长方式，加快调整经济结构，大力推进工业化、城镇化和农业产业化，确保人民生活水平不断提高、社会和谐稳定、国民经济和社会事业更快更好地发展，全面完成2005年及“十五”计划目标。

　　今年全市经济和社会发展的主要预期目标是：全市生产总值增长13%左右，全口径财政收入按可比口径增长12%左右，城市居民人均可支配收入增长10%左右，农民人均纯收入增长7%左右。

　　（一）统筹城乡发展，努力增加县域财力和农民收入。更加注重解决“三农”问题。坚持工业反哺农业、城市支持农村，进一步加大政策扶持、资金投入、科技支持力度，实施工业拉动、项目带动、城乡互动，加快推进农业和农村经济结构战略性调整，努力实现粮食稳定增产、农业持续增效、财力不断增强、农民继续增收。在稳定粮食综合生产能力基础上，坚定不移地调整种植业结构，大力发展园艺特产业和饲料种植业，新建蔬菜生产基地3万亩、绿色食品基地30万亩、无公害农产品生产基地200万亩。继续实施“粮转牧”工程，建设10大优良畜禽基地，新建标准化牧业小区100个，畜牧业增加值增长8%以上。推进东北农业科技创新中心项目建设。加强农村科技推广体系建设，推广测土施肥等30项先进适用技术。创新农业产业化经营模式，全力培育规模大、带动力强的龙头企业。鼓励发展各种形式的新型合作组织，办好产地型批发市场，发展“订单农业”，提高农民进入市场的组织化程度和农业综合效益。紧紧抓住国内部分地区劳务紧缺机遇，搞好组织发动、技能培训、信息服务，不断优化农民进城就业环境，加速推进农村劳动力转移。农民外出务工经商达到90万人次以上。

　　统筹城乡生产力布局，大力发展县域经济。充分发挥资源优势，加快发展县域工业，增强县域经济发展活力。按照集约化发展方向，推进小城镇建设，努力形成比较优势和产业特色。进一步发挥城市辐射带动作用，鼓励城区工业向农村梯度转移，把农产品加工项目尽量摆放在县城、省级开发区和重点镇。加强农业和农村基础设施建设。新建乡村公路2000公里，全市通油（水泥）路行政村比例达到88%左右。加快实现农村客运站点网络化，新建三级以下乡镇客运站20个。积极推进县城和省级开发区水源工程和污水处理工程。发展旱作经济作物区节水灌溉，新增全程节水灌溉面积10万亩。继续推进农安防病改水，新打深水井114眼，从根本上解决氟病区14万人饮用水源问题。改进农村金融服务，支持农村信用社增加农户小额信用贷款和联保贷款。

　　（二）坚持走新型工业化道路，努力提升工业经济持续增长能力。坚持总量扩张和结构调整并重，加快产业集群和制造业基地建设，努力形成一业突出、多业并举的产业格局。工业增加值增长14%以上。

　　举全市之力支持一汽，加快发展汽车产业。规划建设100平方公里长春国际汽车城，为汽车工业发展提供足够空间。依托一汽，整合汽车产业资源，大力引进、发展整车及零部件企业，向城区西南方向集聚。实施丰田发动机、大众汽车线束、旭阳轿车内饰、富奥转向器等45个投资超亿元的重点项目，努力提高整车市场竞争力和零部件配套能力。大众开迪、丰田混合动力车等6种新车投放市场。全力支持一汽主辅分离。积极申报国家汽车零部件出口基地。

　　大力发展农产品加工业。发挥农业资源优势，积极推进隆迪60万吨玉米乳饮品、皓月肉牛开发等21个投资超亿元的重点项目建设，做大做强玉米产业，加快形成以大成为龙头的农产品加工产业群。加快发展肉鸡、生猪、肉鹅等农产品加工体系，建设绿色食品加工基地。农产品加工业产值增长25%以上。积极申报国家农产品加工基地。

　　加快发展光电子信息产业。围绕液晶显示、汽车光电子等五大领域，加快推进北彩4.5代生产线、华信光收发模块、一光编码器等13个投资超亿元的重点项目，以上游关键器件带动下游整机产业，建设长春光电子国家产业基地。光电子信息产业产值增长20%以上。

　　全力发展生物与医药产业。充分发挥生物与医药领域的技术优势，加快实施长生流感疫苗、吉大通源干细胞等16个投资超亿元的重点项目，发展生物疫苗、基因工程药物、生物中药、玉米生物医药中间体等重点产品，建设长春生物医药国家高技术产业基地。生物与医药产业产值增长20%以上。积极申报国家医药产品出口基地。

　　（三）全力抓好项目建设，增强经济社会发展后劲。把项目建设作为振兴老工业基地的突出任务，切实摆上重要位置。从长春产业发展的客观需要出发，集中全社会力量，齐心协力抓投入、上项目、增后劲，做到大中小项目并举，政府投资、引进外资、民间投资齐上，努力实现固定资产投资增长、新上项目规模和质量的新突破，为经济快速发展提供强有力支撑。全社会固定资产投资增长30%以上。围绕四大支柱产业链的延伸和配套，规划建设一批具有骨干支撑作用的工业项目，加快实现支柱产业集群化和主导产品配套化。围绕城市空间结构调整，超前规划建设一批交通、能源、供水等基础设施项目，进一步提高城市的承载能力。围绕经济与社会协调发展，规划建设一批文化、教育、卫生、体育等社会事业的重点项目，努力提高经济社会协调发展水平。切实加大农业基础设施、科技创新、生态建设以及其它薄弱环节的投资力度，多渠道挖掘、引进、培育重点项目。充分利用国家倾斜政策，积极规划争取一批国债项目。

　　进一步降低投资门槛，简化审批程序，提高办事效率，建立项目建设“绿色通道”。盘活土地存量资源，积极争取新增建设用地，保障项目用地需求。加大投融资体制改革力度，充分利用资本市场融资，积极争取金融机构支持。进一步激活民间投资，加快形成投资自主增长机制。实行市级领导干部重点项目包保责任制，形成强有力的项目推进机制。健全和规范项目法定代表人责任制，确立企业投资主体地位，努力提高项目建设速度和质量。设立投资项目库，策划和推出一批新项目，形成“储备一批、开工一批、投产一批”的良性机制。

　　（四）着力实施“开放带动”战略，努力提高市场化水平和开放度。充分利用振兴老工业基地带来的政策效应，紧紧抓住国内外要素重组、产业转移、跨国直接投资和沿海发达地区资本扩张有利契机，采取有效措施，促进招商引资取得新突破。立足我市产业资源优势和外商投资需求，有针对性地包装重点招商项目，灵活开展叩门招商、委托招商、中介招商。发挥企业招商引资主力军作用，加大国有和国有控股企业招商力度，鼓励民营企业对外合资合作。充分利用优强企业、优质资产、优势资源、优良环境，加大对重要客商、重大项目、重点地区的招商力度，突出抓好日本、韩国、德国等国家和台湾、香港等地区的招商。实际利用外资增长30%以上。以“长三角”、“珠三角”地区为重点，大力开展对内招商。着力吸引国内知名企业在长投资、设立窗口。实际利用内资增长20%以上。坚持以提升自主创新能力为出发点，不断优化利用外资结构，引导外资投向四大主导产业、重大基础设施和现代服务业。

　　调整对外贸易结构，推动外贸出口稳步增长。实施“科技兴贸”和“以质取胜”战略，提高机电产品、高新技术和轻纺产品出口比重，努力改变偏重依赖玉米的出口结构。培育建设鹿产品、酱菜等5个重点农产品出口基地，扩大农产品出口。推进企业管理体系和产品质量体系认证及采标工作，使更多的出口企业和出口产品获得“国际通行证”。外贸出口增长10%以上。实施“走出去”战略，加强与东盟、俄罗斯等国家的经贸往来，鼓励有条件企业开展对外投资和跨国经营，带动商品、技术和劳务出口。对外承包工程额和外派劳务均增长10%以上。加强与东北老工业基地重点城市的联合协作，努力拓展国内市场。

　　充分发挥开发区对外开放的辐射带动作用。加快构筑开发区新型行政管理体制，剥离富余人员，推进内部机构和人事、分配制度创新，形成人员能进能出、干部能上能下、工资能高能低的竞争机制，提高运转效率，再造加快发展的生机与活力。动员全市上下为开发区加快发展创造良好的外部环境。建立科学的经济指标评价体系和社会保障、财政支撑体系，合理调整各开发区功能定位和产业分工，提高引进项目质量，推动产业结构升级，促进开发区二次创业。开发区引进超5000万元大项目100个以上，其中超亿元的50个以上。生产总值增长22%以上，占全市比重达到50%，实现2000年提出的经济上“再造一个长春”目标。

　　（五）全力推进改革不断深入，在创新中增强经济自主增长能力。本着“突出重点、积极推进、稳步实施”的原则，积极深化各项改革，切实解决影响全局的深层次矛盾和问题。按照整体改制、债权债务处理、职工身份理顺和国有资本退出到位的要求，坚定不移地推进国企改革攻坚战，基本完成国企改革任务。围绕有利于促进企业发展、维护职工利益、充分调动参与改革者积极性，用好用足国家政策，多渠道筹措资金，解决改革成本缺口问题，在国有职工身份理顺和债务化解等难点上实现新突破。积极支持和推进中、省直国有企业改革。进一步加强国有资产监管，建立并实行年度经营责任制和任期经营业绩考核责任制，防止国有资产流失。加快建立现代企业制度，完善法人治理结构。继续推进事业单位改革。深化财政管理体制改革，构建公共财政框架。加快国有粮食购销企业富余人员分流，推进以企业经营机制转换和产权制度改革为核心的粮食流通体制改革。巩固农村税费制度改革成果，进一步健全减轻农民负担的长效机制。按照“依法、自愿、有偿”原则，稳步推进农村土地使用权流转。推进征地制度改革，确保失地农民不低于原来生活水平。积极推进乡镇机构改革，合理调整乡镇规模，建立服务型乡镇管理体制。

　　大力发展民营经济。树立亲商、富商、安商、扶商观念，营造尊重财富、崇尚创业的致富氛围。引导城乡群众转变创业观念，多渠道增加收入、积累财富。积极打造促进民营经济发展的信息服务、科技创新、项目推进、职工培训等社会化服务平台，为民营经济发展营造良好环境。多渠道筹集担保资金，多形式建立担保机构，努力解决民营企业融资难问题。认真落实各项扶持政策，加快发展中小企业，形成一批强、精、专的中小企业群。支持优势非公有制企业以控股、收购、租赁、联合等方式，参与国有和集体企业的改制改组。鼓励民营企业制度创新、管理创新和技术创新，向外向型、科技型和产业集群方向发展，推动民营经济上规模、上水平。民营经济增加值和实缴税金均增长25%以上。

　　（六）坚持“以人为本”，进一步完善就业和社会保障体系。始终把解决人民群众困难作为各级政府的重大职责和任务，努力为群众办实事、办好事，解决关乎民生的大事、难事。把扩大就业放在经济社会发展更加突出的位置，坚持在发展中解决就业问题，逐步形成有利于扩大就业的经济结构和增长模式。注重发展劳动密集型产业、中小企业和服务业，形成更多的就业增长点，新增就业岗位9万个。认真落实各项再就业政策，鼓励下岗失业人员通过各种方式实现就业。安置下岗失业人员5.8万人，其中“4050”人员9000人。加强就业指导和服务，大力开展职业技能培训，免费培训下岗失业人员2.2万人。城镇登记失业率控制在5%以内。高度重视高校毕业生就业和复员转业军人安置。完善社会保障体系，扩大社会保险覆盖面。抓好非公经济组织和事业单位参保工作，逐步将符合条件的城镇从业人员和失地农民纳入社会保险范围。注重解决国企改制职工社会保险关系接续问题，基本完成国企下岗职工基本生活保障向失业保险并轨。强化养老、失业保险基金征缴，征缴率达到92%以上，确保按时、足额发放。基本医疗保险、工伤保险分别新增5万人和10万人。

　　加强社会救助体系建设。实施敬老院三年改造工程，改善孤寡老人生活条件。落实帮困助学制度，力争不使一个学生因贫失学。落实大病救助制度，努力解决特困群众看病难问题。认真做好城市低保工作，做到应保必保。落实廉租房救助制度，人均住房建筑面积不足8平方米的补贴到14平方米。力争全部解决城区历史遗留的超期回迁问题。继续实施扶残助业工程，安置残疾人就业2200名。深入实施妇女儿童发展规划，办好妇女儿童和残疾人法律援助中心，扩大法律援助覆盖面。动员和依靠社会力量开展帮扶救助活动。

　　健全正确处理人民内部矛盾工作机制。完善信访工作责任制，综合运用政策、法律、经济、行政等手段和教育、协商、调解等方式，及时处理信访问题，妥善处置群体性事件。落实安全生产措施和责任追究制，坚决杜绝恶性事故发生。坚持打防结合、预防为主，加强社会治安综合治理，依法打击各种犯罪活动，营造安全社会环境。发挥城乡基层自治组织和各类民间组织、社会中介组织作用，形成社会管理和服务合力。注重城市安全，健全和完善城市安全总体预案，形成各种突发事件应急机制，提高保障公共安全和处置突发事件能力，做好防灾减灾工作。

　　（七）大力发展服务业，努力扩大消费需求。发展各类金融机构，鼓励金融服务和金融产品创新，加强金融风险防范，促进金融业在规范中快速发展。市商业银行完成增资扩股。大力发展信息服务和中介组织。积极发展房地产业，合理调整结构，规范开发建设秩序，引进知名房地产开发企业，加快城中村和棚户区改造。房地产开发450万平方米，竣工230万平方米，改造棚户区15万平方米。深化住房制度改革，降低二手房交易成本，培育发展住房二级市场。继续加大住房公积金归集与运用力度，扩大覆盖面。启动建设空港物流园区、香江远达和长粮农产品物流中心、东湖物流配送中心，提升汽贸、铁北、东站三大物流园区发展水平，积极发展第三方物流企业，壮大物流产业。

　　下力气培育发展会展旅游业。培育办展主体，优化办展环境，精心办好第4届汽博会、第20届国际光学大会、光电博览会、教育展、冰雪节、电影节等重点展会，举办会展活动120次以上。筹备好世界冬季城市市长会议。整合旅游资源，完善旅游设施，丰富旅游产品。长影世纪城一期、净月潭高尔夫球场投入使用，启动汽车文化园、伪满皇宫三年恢复配套工程。加大旅游促销力度，积极开拓国内外市场，争创“中国最佳旅游城市”。

　　积极鼓励扩大消费。完善《城市商业网点规划》，调整商业网点布局，注重新建区网点建设，方便群众购物。集中培育重庆路、大马路、红旗街、桂林路等商业中心区，发展长江路、建设街、岳阳街路等17条特色商业街，新建续建盛世中华、兽药饲料批发等6个万米以上购物广场和大型专业市场，发展电子商务、连锁经营等新型业态和服务方式，增强现代商业集聚、辐射能力。完善消费政策，积极培育信息、汽车、文化、健康等新的消费热点。大力开拓农村市场，挖掘农村消费潜力。继续整顿和规范市场秩序，深入实施食品、药品放心工程，坚决打击假冒伪劣、欺诈经营等行为，保护消费者利益，改善消费环境。全社会消费品零售总额增长12%左右。

　　（八）坚持规划引导与建设管理并重，不断提升城市的综合竞争力。按照“积极而为，适度超前，量力而行，可持续发展”要求，坚持新区建设与老区改造相结合，规划、建设、管理好城市，努力创造优良人居环境，不断推动人与自然相和谐。高起点编制城市发展规划。认真做好“十一五”规划编制工作，深入研究事关长春发展的全局性、战略性问题，努力提高规划的预见性、科学性和指导性。继续抓好新一轮城市总体规划、土地利用总体规划报批和执行工作。编制城市分区规划、控制性详细规划、城市设计和县级市总体规划，形成完善的规划体系。积极启动南部新城建设。

　　高标准建设城市基础设施。新建北人民大街等6条街路和长新桥等2座桥梁，续建景阳大路等11条街路，大中修45条主次干道，综合改造150条巷道和2个城市出入口，整修道路50万平方米。建设凯旋路客运站，抓好长双烟铁路建设，改建长平一级、科铁线等5条公路。抓紧轻铁二期的申报及建设。龙家堡机场建成并正式通航。实施热电一、二厂二期改造，抓紧热电三厂建设前期工作，推进华能九台坑口电厂项目进程。改造燃气管网60公里、供水管线50公里，综合整治二次供水设施400处。解决市区105条背街暗巷照明问题。新建、改建公厕180座。

　　高效能开展环境综合整治，加强城市管理。深入开展创建国家卫生城活动。扩建三道垃圾场，启动韩家瓦房垃圾处理场建设。实施溪园清淤还湖和长春公园污水处理工程，争取启动北郊污水处理厂二期和南部污水处理厂建设，建设粪便无害化处理厂。深入开展爱国卫生运动，对铁路沿线、城乡结合部等重点部位进行综合整治，1/3以上社区达到“卫生先进社区”标准。加强乡镇市容环境整治，加快农村改水改厕步伐。加大城市管理行政执法力度，切实提高火车站前广场等重点部位的管理水平。巩固畅通工程成果，强化交通秩序管理。

　　大力发展循环经济，加快建设节约型社会。积极引导全社会树立节约能源、节约资源、保护环境意识，按照减量化、再利用、资源化原则，推进资源节约和综合利用，推行清洁生产和节能技术。支持采用新技术处置可燃废弃物及工业废渣，扩大水泥生产。支持利用煤矸石扩大空心砖生产，促进墙体材料革新与建筑节能。加强洗车、洗浴业和其它行业用水管理，建设节水型城市。强化生态环境监管，严格控制主要污染物排放，抓紧解决威胁人民群众健康安全的环境污染问题。

　　高度重视生态环境建设。加强净月潭国家森林公园保护，加快波罗湖湿地恢复，启动石头口门水库上游湿地保护工程，推进“三北”防护林四期工程建设、城郊生态林和农田防护林更新改造，完成植树造林7000公顷。精心建设60块1000平方米以上的城区绿地和人民大街北段等8条绿化精品街路，续建伊通河城区中段风光带，完善雕塑公园二期，启动裕华园建设，改造南湖公园、儿童公园、胜利公园和西安广场、卫星广场，抓好庭院绿化和小区绿化。城区新增绿地300公顷。

　　（九）落实“科教兴国”战略，大力开发人才资源。充分发挥科教人才优势，推动教育、科技、人才与经济紧密结合。坚持科技先导，推进科技创新。充分利用国内外先进技术资源和政策支持，依托重大工程项目，培育自主创新能力，开发具有自主知识产权的核心技术。继续加大政府科技投入，引导更多社会资金参与重大科技项目建设。加快科技资源共享、科技企业孵化、技术交易等科技中介服务体系建设，推进产学研紧密结合，加快高技术重大项目和科技成果的开发、转化和产业化。继续办好科技商业计划书大赛、科技项目融资推介活动。高新技术产业产值增长25%以上。加强科技对外交流与合作，推进中俄、中德科技合作基地建设，精心组织“中德光学技术论坛”、“白俄罗斯科技周”活动。加强知识产权保护和科普工作。

　　坚持教育优先，努力办人民满意教育。加强素质教育，推进初中毕业和高中招生制度改革，完善新课程教学，建立科学的学生、教师、学校评价体系。切实加强以未成年人为重点的思想道德教育。深化学校内部改革，改善师资结构，提高教师整体素质。优化教育结构布局，改善城乡结合部学校办学条件，新建、改造5所城乡结合部中小学。提高普通高中招生计划内统招生比例。规范教育收费，减轻学生和家长负担。更加重视农村义务教育。继续采取加大政府投入、组织社会对口帮扶等措施，改造中小学危倒校舍6万平方米。完善农村现代远程教育达标工程，提高农村中小学信息技术教育水平。做大做强职业教育，加强技能型人才培养培训，搞好农村职业教育中心和实践实训基地建设。深入开展高校与地方政府共建活动，支持在长高校加快发展，促进高校为地方经济发展服务。积极支持吉林大学南迁发展。努力发展学前、社区和成人教育，构建终身教育体系，建设学习型城市。

　　营造良好的人才成长环境。全面推进党政、专业技术、企业经营管理和高技能、农村实用人才5支队伍建设，努力做到人才结构调整与产业结构调整相同步、人才资源开发与经济社会发展相协调。加强企业博士后工作站建设，办好海外学人创业园，不断创新引智载体，积极引进急需和紧缺人才、海外留学人员。引进国外专家1500人次、各类人才10000人以上。研究制定留住人才的政策措施，发挥现有人才的作用。加强农村实用人才的培养和选拔，实施“一村一名大学生计划”。实行收入与绩效挂钩，完善技术、知识、管理等要素参与分配制度，激发人才创造活力。构建统一、开放的人才市场体系，鼓励和引导社会力量发展人才中介。开辟国际人才交流渠道，办好中国国际人才市场长春分市场。

　　（十）大力加强文化建设，加快发展各项社会事业。按照“提供精神动力、激发创业热情、提高人的素质、营造和谐家园、塑造长春形象”要求，全面实施“道德弘扬、诚信警示、社会公益、环境营造、创业致富、创建实践”文明长春系列行动，努力提高市民的文明素质和城市的文明程度，力争用2—3年时间，进入全国文明城市行列。巩固“双拥模范城”成果，继续推进以部队信息化建设为重点的科技拥军工作。

　　加快发展文化事业和产业。加大对公益性文化事业的扶持力度。制定改革政策，加快推进经营性文化产业体制和机制创新。积极组织艺术精品演出，举办广场文化活动200场以上，丰富群众文化生活。进一步发展新闻出版和广播影视业，提供健康向上、群众喜闻乐见的精神文化产品。继续加强文化市场和互联网的建设与管理，坚持不懈地开展“扫黄”、“打非”。办好首届长春文化艺术节、第三届长春民间艺术博览会、第五届长春图书交流会和第十届长春书市。

　　大力发展卫生事业。加强公共卫生体系建设，逐步健全覆盖城乡、功能完善的疾病预防控制和医疗救治体系，加强非典、人间禽流感、鼠疫、霍乱、艾滋病、结核病及流行性出血热的防治工作，提高应对重大传染病等突发性公共卫生事件的能力。改扩建长春急救中心。进一步加强农村医疗卫生工作，推进卫生监督体制改革，完善农村三级医疗卫生保健网络，恢复乡镇中心卫生院功能，加强村卫生所建设与管理。完善九台新型农村合作医疗制度试点，逐步建立农民医疗互助共济制度。推进城镇医疗卫生体制改革，完善医疗机构分类管理，增强社区卫生综合服务功能。整合医疗卫生资源，吸收社会资金发展医疗卫生事业。稳定低生育水平，人口自然增长率控制在2.2‰以内。加强人口战略研究，实施出生缺陷干预工程，优化人口结构。加强流动人口与计划生育管理和服务。

　　做好亚冬会各项筹备工作。落实《长春2007年亚冬会行动计划》，全面启动市场开发，完成亚冬会比赛场馆建设与改造，开始转入赛前试运行。加强群众体育设施建设，建成2个体育健身园、40条健身路径，创建10个体育特色乡镇。积极承办国际、国内大型体育比赛，承办好吉林省第六届少数民族运动会。开展亚冬会系列宣传活动，提高全体市民参与意识，营造良好氛围。

　　（十一）切实加强民主法制建设，全面提高政府行政能力。按照科学执政、民主执政、依法执政要求，进一步转变政府职能，推进行政体制改革，把主要精力逐步转向经济调节、市场监管、社会管理和公共服务，在实践中努力提高行政能力。

　　积极推进民主政治建设。始终自觉地接受人大法律监督和工作监督，支持政协履行政治协商、民主监督、参政议政职能，认真听取各民主党派、工商联、无党派人士意见，加强与工会、共青团、妇联等社会团体和组织的联系，接受群众监督和舆论监督，不断改进政府工作。加强基层民主政治建设。推进社区建设，完善村民自治，落实村务公开、厂务公开，依法保障人民群众的知情权、参与权、选择权和监督权。

　　深入贯彻《全面推进依法行政实施纲要》，建设法治政府。牢固树立依法治市、依法行政观念，带头维护宪法和法律的权威，严格按照法定权限和程序行使权力、履行职责。切实加强行政立法工作。严格落实执法责任制和执法过错追究制，扩大城市管理相对集中行政处罚权范围，认真解决有法不依、执法不公和多头执法、执法扰民等群众反映强烈的问题，努力做到严格执法、公正执法和文明执法。加强行政执法监督，切实做到有权必有责、用权受监督、违法要追究。

　　加快行政管理方式创新。深入推行政务公开，凡不涉及党和国家机密的政务信息均向社会公开，政府所有部门均实现网上信息公开，社区普遍设立政府信息查询点。深化行政审批制度改革，完善政务中心功能，改进基本建设审批程序和办法，努力做到“一口受理、一次收费、并联审批、限时办结”。推进电子政务建设，逐步建立全市统一的电子政务工作体系，完成政府专网升级改造和政务中心联网审批系统，推动政府工作提速提效。

　　加强政风建设。坚持以人为本、执政为民，把维护好、发展好、实现好广大人民群众的根本利益作为政府一切工作的出发点和落脚点。坚持一切从实际出发，按客观规律办事，量力而行，不盲目攀比。坚持办实事，求实效，珍惜民力，不搞劳民伤财的“形象工程”。坚持察实情，讲真话，不虚报浮夸。倡导扎实作风，精简会议，减少文件，把更多时间和精力用于深入基层，解决实际问题。

　　深入治理软环境。针对软环境建设中存在的突出问题，强化治本措施，建立长效机制。继续清理行政事业性收费，降低企业和群众负担。拓展软环境建设范围，开展检验、检疫、鉴定、签证等领域的专项治理。发挥软环境投诉举报中心作用，做到有诉必查、有错必纠、查实必处。健全公务员激励机制和监督约束机制，继续开展民主评议政行风和“百名处长”活动、评选人民满意公务员和集体活动，并将结果作为奖惩、任用和淘汰的重要依据。对权力集中的行政岗位负责人进行定期交流。重视和加强公务员培训和考核，切实提高公务员素质，努力建设政治坚定、业务精通、清正廉洁、作风优良的公务员队伍。加强对权力运行的监督和管理，严格执行经济责任审计制度，支持监察、审计部门依法独立履行监督职责。坚决纠正各种损害群众利益行为，重点解决土地征用、房屋拆迁、企业重组改制和破产等工作中存在的问题。坚持不懈地开展廉政建设和反腐败斗争，建立健全“三位一体”的反腐倡廉整体工作格局，提高行政监察、效能监察能力，坚决查处违纪违法案件，惩处腐败分子。

　　各位代表，长春面临的机遇与挑战并存，振兴老工业基地的历史使命光荣而艰巨。让我们更加紧密地团结在以胡锦涛同志为总书记的党中央周围，在市委的正确领导下，在人大、政协的有效监督和支持下，调动一切积极因素，凝聚加快发展的强大动力，以只争朝夕的精神，努力开创各项工作新局面，为早日实现全面小康社会目标，早日让全市人民过上幸福安康生活而努力奋斗！
